# Identification of microRNA expression profiles in the gill, intestine and hepatic caecum of *Branchiostoma belcheri*

**DOI:** 10.1007/s13238-016-0365-3

**Published:** 2017-01-23

**Authors:** Xin Liao, Liu Yang, Xi Chen, Junyuan Chen

**Affiliations:** 1 School of Life Sciences, Nanjing University, Nanjing, 210093 China; 2 Beihai Marine Station of Evo-Devo Institute, Nanjing University, Nanjing, 210093 China; 3 Department of Biomedical Research Center, The First People’s Hospital of Kunming, Kunming, 650011 China; 4 Nanjing Institute of Geology and Paleontology, Nanjing, 210008 China; 5 State Key Laboratory of Pharmaceutical Biotechnology, Jiangsu Engineering Research Center for MicroRNA Biology and Biotechnology, NJU Advanced Institute for Life Sciences (NAILS), School of Life Sciences, Nanjing University, Nanjing, 210093 China

**Dear Editor,**

The origin of vertebrate-specific characters and functional systems is one of the most important questions in the evolutionary biology (Holland and Chen, 2001). Amphioxus, the most basal chordate, is considered as the sister of vertebrates based on the recent molecular phylogenetic evidences, and it is used as an ideal model organism to study the origin and evolution of vertebrate organ systems including immune system (Yu and Holland, 2009). Three digestive organs, gill slits, hepatic caecum and intestine in amphioxus were also considered as primitive immune organs of amphioxus (Schmitz et al., 2000). In addition to the respiratory and digestive function, the amphioxus gill is regarded as “the first immune defense organ” and plays a natural immune defense role by working as a physical barrier. The intestine is serving as a site of continuous immunological interaction (Yuan et al., 2015). Meanwhile, the hepatic caecum of amphioxus, as a prototype of the vertebrate liver, is also considered as a potential immunological organ in the amphioxus, playing an important role in acute phase response (APR) (Wang and Zhang, 2011). The immune responsive cells including lymphocyte-like cells and macrophage-like cells were found in the gill and intestine of amphioxus, respectively (Huang et al., 2011). Furthermore, the conserved molecules and signaling pathways involved in innate immune system were also identified in these organs in the amphioxus (Huang et al., 2011). Though the importance of these organs in digestive system and the value of these tissues for study of immune system evolution are demonstrated (Yuan et al., 2015), the molecular characters of these tissues are not revealed comprehensively.

To date, whole genome have been sequenced for two amphioxus species *B. floridae* and *B. belcheri*, and tissue-specific transcriptome of *B. belcheri* have been sequenced recently (unpublished, http://wcy.pkusulab.com). Meanwhile, a number of researches around noncoding RNAs in amphioxus were published. The miRNAs are a class of endogenous noncoding RNAs with approximately 22 nucleotides, and play important role in post-transcriptional regulation of gene expression. The global expression of miRNAs in the whole amphioxus is being revealed (Chen et al., 2009). However, the expression pattern of miRNAs in the amphioxus is not yet discovered. As a starting point to fill in this knowledge gap, here we investigate the expression profiles of miRNAs in the three digestive and immune-related organs: gill, intestine and hepatic caecum.

To reveal the expression patterns of miRNAs in these organs, we utilized a μParaflo-microfluidic chip-microarray screen performed by LC Sciences (Hangzhou, PR China). In two independent replicates, we calculate the number of miRNAs with microarray signal intensity greater than 50 in each organ based on 272 target probes from *B. belcheri* and *B. floridae*, and identified 138 miRNAs uniquely expressed in the gill, 127 miRNAs in the intestine and 107 miRNAs in the hepatic caecum, 114 miRNAs in both gill and intestine, 53 miRNAs in both gill and hepatic caecum, 45 miRNAs in both intestine and hepatic caecum, and 42 miRNAs in all three organs, a Venn diagram is shown in Fig. S1. The miRNA microarray data were deposited to NCBI Gene Expression Omnibus (GEO) database under accession number GSE87094. To overview the global expression patterns of miRNAs in these three organs, we generated a heatmap including all miRNAs in our microarray chip (Fig. 1A). Further analysis of the heatmap showed eight different expression trends of miRNAs in these three organs (Fig. 1B–I). These different expression trends of miRNAs suggest a dynamic expression profile of miRNAs in distinct organs. Interestingly, the hierarchical cluster and statistical analysis showed that the miRNA expression pattern of gill and intestine is close to each other as compared to the expression pattern of hepatic caecum and intestine or the expression pattern of hepatic caecum and gill. The previous reports indicated that several innate immune-related genes have parallel expression pattern in the gill and the intestine before and after immune stimulation (Yuan et al., 2009), which may suggest that these two organs act as the first line of immune defense and share responsibility for immune response. On the other hand, the hepatic caecum is also considered as part of the digestive system and involved in immune responses. But, hepatic caecum primarily acts as a homological organ of liver, which is different to the gill or the intestine. Thus, it’s not surprise that the miRNAs in hepatic caecum has a relative different expression pattern as compared to the gill or the intestine.

**Figure 1:**
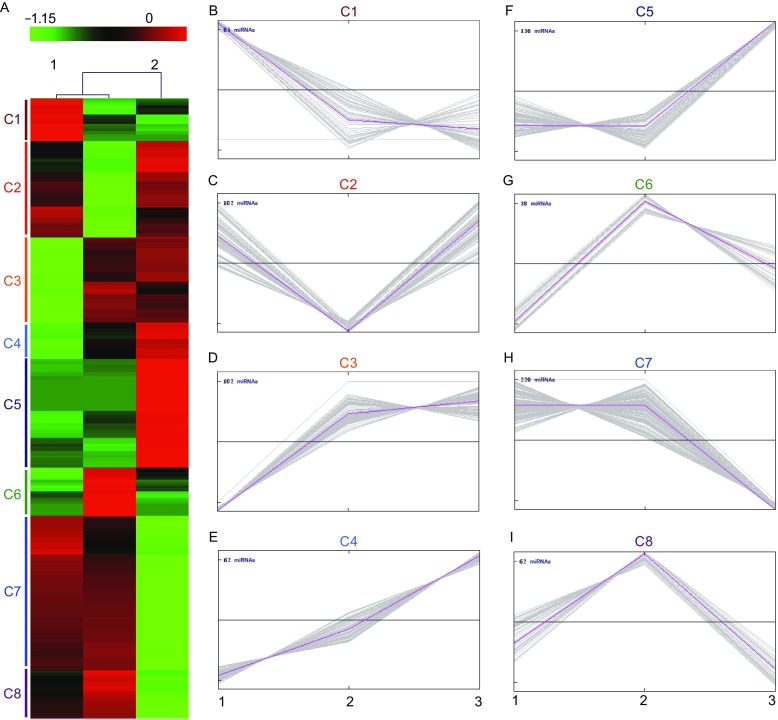
**The expression profile of miRNAs in different organs of amphioxus.** (A) Heatmap illustrates the expression levels of miRNAs in the intestine (1), the gill (2) and the hepatic caecum (3). The expression levels were represented by a color scale with green as the lowest expression and red as the highest expression. Based on the expression profile of miRNAs, the hierarchy cluster analysis was applied and eight different expression trends were labeled as C1 to C8. (B–I) The eight expression trends (C1 to C8) as indicated in (A) were plotted with the expression values of individual miRNAs. The horizontal line in the middle indicates the middle expression level. The miRNAs above the line are considered as high expression levels and the miRNAs below the line are considered as low expression levels. The expression levels of miRNAs are: 1) high in the intestine but low in the gill and hepatic caecum (B); 2) high in both the intestine and hepatic caecum (C); 3) low in the intestine but high in both the gill and hepatic caecum (D); 4) low in the intestine, medium in the gill and high in the hepatic caecum (E); 5) low in both the intestine and gill but high in the hepatic caecum (F); 6) low in the intestine, high in the gill and medium in the hepatic caecum (G); 7) high in both the intestine and gill, and low in the hepatic caecum (H); 8) medium in the intestine, high in the gill and low in the hepatic caecum (I)

To further explore the expression pattern of miRNAs, the difference of expression levels of miRNAs in paired organs was compared. The expression levels of miRNAs with significant differences in two organs are shown in Table S2. Our analysis showed that there are 67 miRNAs with higher expression levels and 47 miRNAs with lower or no expression in gill as compared to the hepatic caecum (Table S2). Meanwhile, 45 miRNAs are abundantly present and 49 miRNAs are poorly expressed in hepatic caecum as compared to the intestine (Table S2). Interestingly, there are only 15 miRNAs with higher expression and 13 miRNAs with lower or no expression in the gill as compared to the intestine (Table S2).

For those miRNAs with significant expression difference, we then focused on miRNAs identified from *B. belcheri* (*bbe*) and *B. floridae* (*bfl*). This analysis showed that 20 miRNAs expressed higher and 11 miRNAs lower in the gill as compared to the hepatic caecum (Fig. 2A). Eight miRNAs showed abundant expression in intestine as compared to the hepatic caecum and 14 miRNAs are expressed highly in hepatic caecum as compared to the intestine (Fig. 2B). In addition, 2 miRNAs and 4 miRNAs are highly expressed in the gill and the intestine, respectively (Fig. 2C). Taken together, these data demonstrate that there are groups of miRNAs with abundant expression in one tissue but not in another tissue, which may suggest the probability of existence of tissue-specific miRNAs in distinct organs.

**Figure 2:**
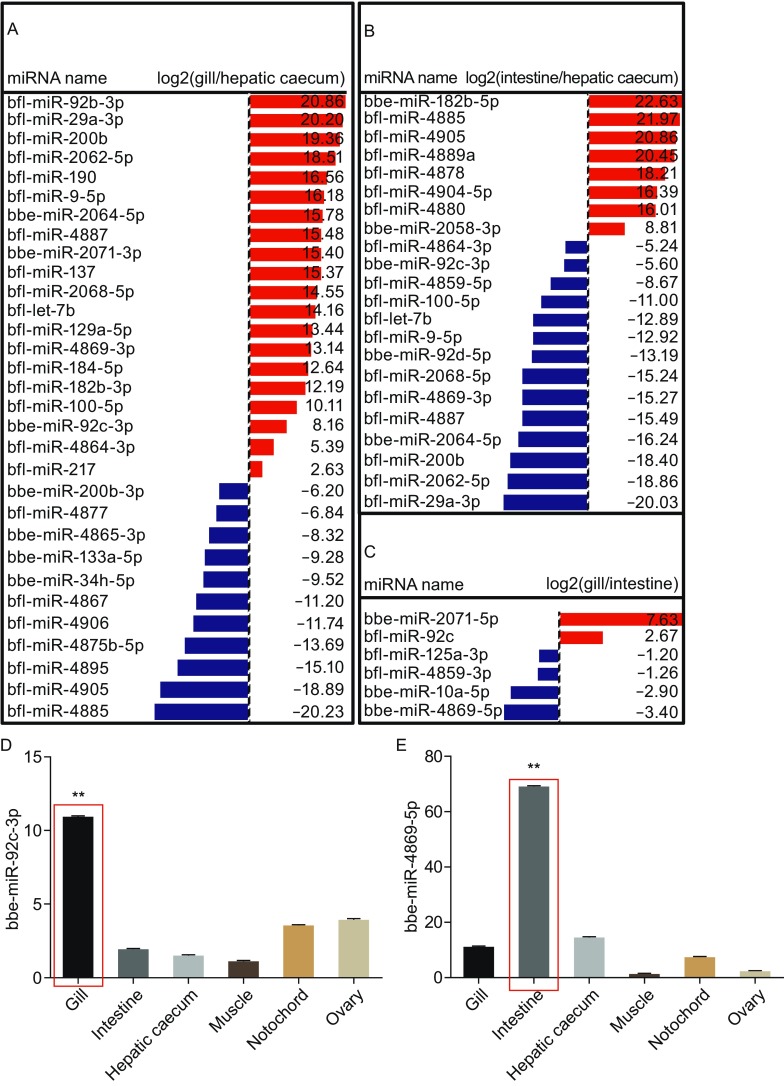

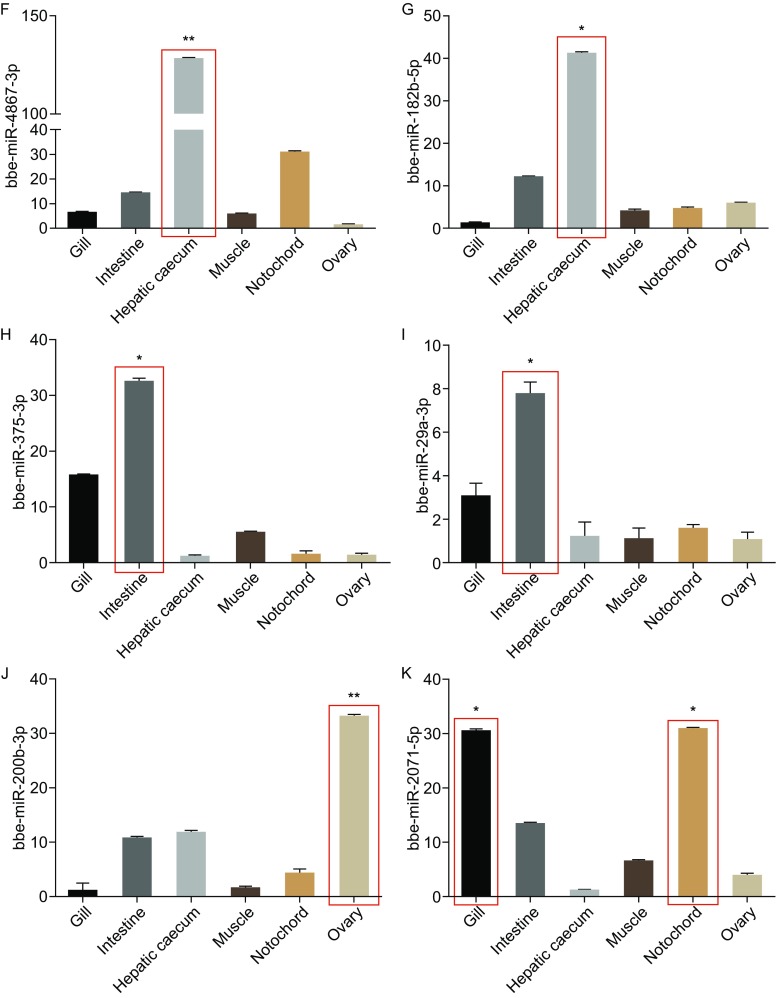
**Statistical analysis the expression difference of miRNAs in paired organs of amphioxus, and validation of the expression of tissue-specific miRNAs.** The relative expression levels of miRNAs identified from the *B. floridae* and *B. belcheri* were statistically compared using the Student’s *t* test. The miRNAs with significant difference in the comparisons of gill versus hepatic caecum (A), intestine versus hepatic caecum (B) and gill versus intestine (C) were shown. The red color indicates high expression of miRNAs in the organs listed as the numerator and the blue indicates high expression of miRNAs in the organ listed as the denominator. The quantitative PCR results of the expression of the following miRNAs in the gill, intestine, muscle, hepatic caecum, notochord and ovary were shown: bbe-miR-92c-3p (D), bbe-miR-4869-5p (E), bbe-miR-4867-3p (F), bbe-miR-182b-5p (G), bbe-miR-375-3p (H), bbe-miR-29a-3p (I), bbe-miR-200b-3p (J) and bbe-miR-2071-5p (K). The data are expressed as a ratio to the expression level of U6 and are plotted as the means ± SD. (*P < 0.05, **P < 0.01)

Next, 18 miRNAs with significant expression difference were selected for further validation using quantitative RT-PCR. These miRNAs are identified from *B. belcheri* or *B. floridae* in the microarray screen, and since *B. belcheri* and *B. floridae* are closely relative animals from one genus, a part of miRNAs share identical sequences (88 miRNAs sharing identical sequences between the two species in miRBase release v21.0, see details in Table S3), then we unified the IDs to *B. belcheri* miRNA IDs when the selected miRNAs share identical sequences between the two species (Table S3). We examined the expression levels of selected 18 miRNAs in six different tissues from *B. belcheri* including the gill, intestine, hepatic caecum, muscle, notochord and ovary (Fig. 2D–K and S2). Interestingly, some miRNAs showed very specific expression pattern in one tissue but not other tissues that are examined here. For example, bbe-miR-92c-3p has a significant high expression in the gill but not in other 5 tissues (Fig. 2D). The bbe-miR-4869-5p, bbe-miR-375-3p and bbe-miR-29a-3p are abundant in the intestine but not others (Fig. 2E, 2H and 2I), and bbe-miR-4867-3p and bbe-miR-182b-5p are highly expressed in the hepatic caecum (Fig. 2F and 2G). Unexpectedly, we also identify a miRNA, bbe-miR-200b-3p, significant with high expression in the ovary (Fig. 2J). In addition, there are also some miRNAs with particular high expression in 2 tissues but not in other 4 tissues. Specifically, bbe-miR-2071-5p is abundant in both the gill and the notochord (Fig. 2K). These results thus provide evidences that some miRNAs could specifically express in a certain tissue among the six tissues we examined.

Among these miRNAs, bbe-miR-92c-3p, bbe-miR-182b-5p, bbe-miR-200b-3p, bbe-375-3p and bbe-miR-29a-3p are well evolutionally conserved among species according to the alignment analysis of their sequences on miRBase website (http://www.mirbase.org/). The homologies of these miRNAs have been found to play important roles in specific tissues. For example, miR-17~92, the homology of bbe-miR-92c-3p, has been implicated in the regulation of respiratory system development including embryonic lung development (Lu et al., 2007), which is consistent with its high expression in the gill of the amphioxus. As a high expression miRNA in the primitive liver of the amphioxus, the homology of bbe-miR-182b-5p has been reported to be involved in liver metastasis, liver injury and inflammation (Blaya et al., 2016). Additionally, although miR-375 was originally reported to be a pancreas islet cell-specific miRNA, it is also expressed in the gastrointestinal organs and abnormally regulated in the gastric cancer and colorectal cancer (Song and Ajani, 2013). Since there is no published data about pancreas-like organ in amphioxus, the expression pattern of bbe-miR-375-3p might give us a hint that the original function of miR-375 is regulating mRNA expression in digestive organs, and then evolved the function of regulating insulin secretion in pancreas. Interestingly, we also found several tissue-specific miRNAs including bbe-miR-4869-5p, bbe-miR-4867-3p and bbe-miR-2071-5p in the context of the six tissues we investigated, are solely expressed in the amphioxus based on miRBase database. These miRNAs may represent miRNAs having transient roles in regulating functions of specific tissues during evolution.

To uncover the potential roles of miRNAs during the evolution of complicated immune system, the role of individual miRNA in the amphioxus is beginning to be explored. For example, it was found that bbe-miR-92d regulates the complement pathway through targeting C3 for controlling the acute immune response to bacterial infections in amphioxus (Yang et al., 2013). In addition, the tissue-expression pattern of C3 correlated well with the pattern of bbe-miR-92, implying a functional association between C3 and miR-92 in the immune organs of amphioxus (Yang et al., 2013). This study emphasizes the role of miRNAs in immune regulation of the amphioxus. Interestingly, a couple of the tissue-specific miRNAs in amphioxus discovered in our study, have also been found to play important roles in immune regulation. For example, hsa-miR-92a-3p (homology of bbe-miR-92c-3p) negatively regulates Toll-like receptor (TLR)-triggered inflammatory response in macrophages (Lai et al., 2013). As the human homology of bbe-miR-29a-3p, hsa-miR-29a-3p promotes lipid uptake during monocyte-macrophage differentiation (Wang et al., 2015). And, the hsa-miR-375-3p (homology of bbe-miR-375-3p) has an important role in epithelium immune system by regulating goblet cell differentiation (Biton et al., 2011). Given their specific expression pattern in the amphioxus, their conservations between amphioxus and human, and the crucial functions of their human homologies in the immune system, it’s reasonable to propose that these miRNAs may participate in shaping the “pre immune system” in the amphioxus, and play conserved roles during immune system evolution.

In summary, this study provides a systemic analysis of the expression of miRNAs in three different digestive organs that are associated with immune system evolution, and identifies a set of miRNAs with tissue-specific expression patterns. These results form a basis to further study the function of miRNAs in specific tissues and during evolution.

## Electronic supplementary material

Below is the link to the electronic supplementary material.

Supplementary material 1 (XLSX 9 kb)

Supplementary material 2 (XLS 67 kb)

Supplementary material 3 (XLSX 43 kb)

Supplementary material 4 (PDF 133 kb)
