# Supplementary material for: Identification of microRNA expression profiles in the gill, intestine and hepatic caecum of Branchiostoma belcheri
Source: Protein Cell. 2017 Jan 23;8(4):302–7. doi: 10.1007/s13238-016-0365-3 (PMC5359183; doi:10.1007/s13238-016-0365-3)
Supplement: Supplementary file 4 — Supplementary material 4 (PDF 133 kb) [file 13238_2016_365_MOESM4_ESM.pdf]

Materials and methods

Animals

Amphioxus (*B. belcheri*) were obtained from the sandy bottom of the sea near Zhanjiang at Guangdong, China, and cultured in Beihai Marine Station of Nanjing University at Beihai, Guangxi, China. They were fed on chlorella and isochrysis with well-aerated circulating filtered seawater at 26 ±2 °C. Fifteen adults of amphioxus were used to harvest the gill, intestine and hepatic caecum tissues, and the tissues were rapidly frozen by liquid nitrogen for further RNA extraction. A total of 2 sets of RNA were extracted for each tissue for further microarray assays. This study did not include endangered or protected species. Animal welfare and experimental procedures were carried out in accordance with the Guide for the Care and Use of Laboratory Animals (Ministry of Science and Technology of China, 2006) and the related ethical regulations of Nanjing University. The animal experiments in this study were approved by Nanjing University Animal Care and Use Committee and were arranged to minimize suffering and to reduce the number of usage of animals.

RNA extraction and microarray experiments

Total RNA for each organ was extracted using trizol reagent (Invitrogen, Carlsbad, CA, USA) according to the manufacturer's protocol. The microarray experiments and following data analysis for microarray results were performed by LC Sciences (Hangzhou, PR China). In brief, the assay initially used 4 to 8 μg total RNA sample to generate a poly(A) tail by 3'-extension using poly(A) polymerase. Then, an

oligonucleotide tag was ligated to the poly(A) tail for later fluorescent dye staining. Next, hybridization of RNA samples to microarray probes, which detected miRNA transcripts in the miRBase (release version 21.0, (http://www.lcsciences.com/discovery/applications/transcriptomics/mirna-profiling/mirna/mirna-available-arrays), was performed overnight on a μParaflo-microfluidic chip using a micro-circulation pump (Atactic Technologies) (Gao et al., 2004). The hybridization was conducted in 100 L 6X SSPE buffer (0.90 M NaCl, 60 mM Na_2HPO_4, 6 mM EDTA, pH 6.8) containing 25% formamide at 34 °C. After RNA hybridization, tag-conjugating Cy3 dye were circulated through the microfluidic chip for dye staining. Fluorescence images were collected using a laser scanner (GenePix 4000B, Molecular Device) and digitized using Array-Pro image analysis software (Media Cybernetics). The detected miRNA transcripts include 50 control probes and 1686 miRNAs in miRBase 21.0 from 11 species: 93 of *Branchiostoma belcheri*, 167 of *Branchiostoma floridae*, 7 of *Xenoturbella bocki*, 543 of *Ciona intestinalis*, 10 of *Ciona savignyi*, 62 of *Oikopleura dioica*, 274 of *Petromyzon marinus*, 104 of *Saccoglossus kowalevskii*, 60 of *Strongylocentrotus purpuratus*, 250 of *Danio rerio* and 116 of *Xenopus tropicalis,* and each probe repeated twice.

Microarray data analysis

The raw florescent signal intensity was subtracted from the background signals and was normalized using a LOWESS filter (Locally-weighted Regression) (Bolstad et al., 2003). The heatmap with hierarchical clustering analysis and the expression tendency of miRNAs generated with the normalized data by using software MeV (v4.9,

http://www.tm4.org/mev.html). In addition, the normalized signals were used to compute Z value using the following equation:

$$Z\ sample - i = \frac{log2(Signalsample - i) - Mean\ (Log2(Signal)\ of\ all\ samples)}{Standard\ deviation\ (Log2(Signal)\ of\ all\ samples)}$$

According to the Z values, the expression difference of miRNAs was compared and tested using student's t-test. The significance threshold was set to be 0.01.

Consolidated Microarray data

173 miRNAs of *B. belcheri* and of 187 of *B. floridae* are including in miRBase release version 21.0, while, 88 miRNAs share identical sequence between the two species, thus, there are 272(173+187-88) unique miRNAs from *B. belcheri* and *B. floridae* in miRBase release version 21.0. All of the 272 miRNA probes were performed in our microarray, while a part of them were named to other species in the microarray because of the identical sequences. We tabulated all of the miRNAs in miRBase release version 21.0 from *B. belcheri* and *B. floridae*, marked the miRNAs sharing identical sequences, and list the microarray signal reads of 272 unique miRNAs in Supplementary Table 3.

Quantitative real-time PCR

The preparation of RNA samples from the gill, intestine, hepatic caecum, muscle, notochord and ovary tissues was described above. To further verify the specific expression of miRNAs in different tissues, 18 miRNAs were subjected to stem-loop qRT-PCR. Stem-loop qRT-PCR amplifications were carried out using stem-loop RT primer, forward primer and universal reverse primer. 100 ng of total RNA was reverse

transcribed to cDNA with stem-loop RT primer of each miRNA using AMV Reverse Transcriptase (TaKaRa, Dalian, China) in a 10 µl reaction system consisting of 1 µl RNA template, 0.5 µl RT primer, 2 µl of $MgCl_2$, 0.5 µl AMV RT Enzyme, 1 µl PCR buffer, 1 µl dNTP Mixture and RNase-free double-distilled water. Reaction conditions were 30 °C for 10 min, 42 °C for 30 min, 99 °C for 5 min and 4 °C for 5 min. Then, qRT-PCR was carried out in reaction mixtures comprising 2 µl cDNA, 0.4 µl miRNA-specific forward primer, 0.4 µl universal reverse primer, 10 µl of 2X SYBR *Premix Ex Taq* (TaKaRa, Dalian, China) and 7.2 µl distilled water. The qRT-PCR protocol was as follows: 95 °C for 30 s, followed by 40 cycles of 95 °C for 5 s and 60 °C for 31 s on an ABI 7500 Sequence Detection System. All of the reactions were performed in triplicate. The U6 miRNA was used as an internal control for miRNA expression. The values for the miRNA expression were acquired using the ABI 7500 Sequence Detection System and analyzed using the Software v2.0.1 (Applied Biosystems, Foster City, CA, USA). The primers used for PCR experiments were synthesized by Sangon Biotech (Shanghai, China) and listed in supplementary table 1.

Statistical analysis

The data were presented as the mean ± SD of at least three independent experiments. The compared statistics were performed using two-tailed Student t test. The differences were considered statistically significant when $p < 0.05$.

Figure S1. Venn diagram of number of expression miRNAs in three organ, Validation of the expression of other miRNAs in different tissues.
Figure S2. The quantitative PCR results of other interesting miRNAs in the gill, intestine, muscle, hepatic caecum, notochord and ovary were shown. The miRNAs

that were examined were indicated in the graphs. The data are expressed as a ratio to the expression level of U6 and are plotted as the means ± SD.

Table S1. The primers used for quantitative RT PCR experiments.

Table S2. Statistical comparisons of miRNA expression levels between organs of microarray result, $p<0.01$

Table S3.

Sheet 1, mature miRNA IDs of *B. belcheri* and *B. floridae* in miRBase v21.0, the identical sequences between these two species are marked pink.

Sheet 2, microarray data of 272 miRNAs probes from *B. belcheri* and *B. floridae*.

Sheet 3, list of selected miRNAs' sequences, target names in microarray and corresponding names in *B. belcheri*.
